# Alpha-Catulin, a New Player in a Rho Dependent Apical Constriction That Contributes to the Mouse Neural Tube Closure

**DOI:** 10.3389/fcell.2020.00154

**Published:** 2020-03-17

**Authors:** Kamila Karpińska, Christine Cao, Vicky Yamamoto, Mateusz Gielata, Agnieszka Kobielak

**Affiliations:** ^1^Laboratory of the Molecular Biology of Cancer, Centre of New Technologies, University of Warsaw, Warsaw, Poland; ^2^Department of Otolaryngology-Head and Neck Surgery, Norris Comprehensive Cancer Center, Keck School of Medicine, University of Southern California, Los Angeles, CA, United States

**Keywords:** apical constriction, apical polarization, Rho (Rho GTPase), catulin, actomyosin

## Abstract

Coordination of actomyosin contraction and cell-cell junctions generates forces that can lead to tissue morphogenetic processes like the formation of neural tube (NT), however, its molecular mechanisms responsible for regulating and coupling this contractile network to cadherin adhesion remain to be fully elucidated. Here, using a gene trapping technology, we unveil the new player in this process, α-catulin, which shares sequence homology with vinculin and α-catenin. Ablation of α-catulin in mouse causes defective NT closure due to impairment of apical constriction, concomitant with apical actin and P-Mlc2 accumulation. Using a 3D culture model system, we showed that α-catulin localizes to the apical membrane and its removal alters the distribution of active RhoA and polarization. Actin cytoskeleton and P-Mlc2, downstream targets of RhoA, are not properly organized, with limited accumulation at the junctions, indicating a loss of junction stabilization. Our data suggest that α-catulin plays an important role during NT closure by acting as a scaffold for RhoA distribution, resulting in proper spatial activation of myosin to influence actin-myosin dynamics and tension at cell-cell adhesion.

## Introduction

Actomyosin contractility in epithelia with actin filaments, along with myosin and Rho GTPases, plays a role in changing tissue shape during development. Forces generated using this machinery are transmitted from cell-to-cell through adherens junctions (Arnold et al., 2017). Well-coordinated across a field of cells, actomyosin contractility plays a crucial role in multistep tissue morphogenetic processes, like the formation of the neural tube (NT). This process is regulated by more than 300 genes in mammals (Sawyer et al., 2010; Wilde et al., 2014) and is important for human health, as neural tube defects are among the most common human congenital malformations (Mitchell, 2005). Neurulation involves three-dimensional changes in the shape of the neural plate, where connected neuroepithelial cells undergo coordinated dynamic reorganization, maintaining cell-cell junctions. This is controlled spatially and temporarily by rearrangement of cytoskeleton. Folding of the neural tube occurs through bending at several hinge points that run along the length of the forming tube. The hinge points are the site of columnar epithelial cells undergoing extensive shape change called apical constriction that transforms them into wedge shape by reducing its apical surface area, aiding in the bending of the neural plate (Nikolopoulou et al., 2017).

Integration of two key machineries, namely force generating actin-myosin cytoskeleton and cell-cell adhesion, is crucial for cell shape changes as it links cytoskeleton to the cell membrane and other cells (Martin and Goldstein, 2014; Jodoin et al., 2015; Priya and Yap, 2015). Adherens junctions are known sites of actin filament growth by both the branched actin nucleator Arp2/3 and formin, and this growth stabilizes junctions and supports epithelial tension (Kobielak and Fuchs, 2004; Kobielak et al., 2004; Buckley et al., 2014; Arnold et al., 2017). Actin filaments, along with phosphorylated Myosin Light Chain (P-Mlc) and Rho GTPases, are found in the apical junctions and apical membrane of neuroepithelial cells (Kinoshita et al., 2008; Escuin et al., 2015; Rolo et al., 2016). To establish the contractile actomyosin network, Rho GTPase activity has to be tightly regulated spatially and temporarily. It is well accepted that the placement and extent of GTPase activity is the result of the net activity of guanine nucleotide exchange factors (GEFs) and GTPase-activating proteins (GAPs). It was first thought that apical constriction might occur by contraction of the pool of actin and myosin at cell-cell junctions, resulting in the closure similar to a purse string. However, it was later shown that apical constriction actually occurs in a step-wise fashion, involving pulses of contractions in the medioapical actomyosin network, pulling the adherens junctions inwards (Martin et al., 2009; Teo and Yap, 2016). While it is well established that adherens junctions are required for the transmission of force across an epithelium (Martin et al., 2010; Arnold et al., 2017), it is not clear how actomyosin cortex, which spans the apical surface of an epithelium, transitions between elongation and nucleation of actin or how it involves in the stable attachment of adherens junction. Work from Jodoin et al., indicated that apical constriction can be achieved by an actomyosin cortex spanning the apical surface with rapid turnover that is essential to couple the contractile actomyosin network to adherens junctions, ultimately allowing balanced forces to be stably propagated across tissues (Martin and Goldstein, 2014; Jodoin et al., 2015).

While these recent data raised many challenging questions, one key unsettling question is how the dynamic actomyosin network assembled and remodeled with the transition from state of elongation versus active actin nucleation while remaining connected to cell junctions. The detailed components responsible for the process also remain obscure. Since the actomyosin machinery has to be located at the right place and time to generate required force to pull the neural folds together (Sawyer et al., 2010), it is important to characterize scaffold proteins that may be important for directing Rho family GTPase signaling during neurulation. α-catenin family member, α-catulin (CTNNAL1), is a cytoskeletal linker protein that has been shown to be important in inflammation, apoptosis and cytoskeletal reorganization (Park et al., 2002; Wiesner et al., 2008). Previously, we have demonstrated that α-catulin is upregulated and preferentially expressed at the tumor invasion front and in the invasive streams of cells in malignant human head and neck squamous cell carcinomas (hHNSCC), with minimal expression in the normal oral epithelia. Decrease in α-catulin expression in cancer cells results in diminished cell migration and invasion *in vitro* and reduced tumor metastasis *in vivo*, in a xenograft mouse model (Cao et al., 2012). α-catulin was also shown to be highly expressed in malignant melanoma cells compared to melanocytes and be a crucial driver of tumor progression, invasion and metastasis (Kreiseder et al., 2013). This protein has not been extensively characterized so far, but interestingly, it was shown to directly interact with Lbc-Rho GEF and enhance Rho GTPase signaling, acting as a scaffold for the Rho GTPase signaling complex (Park et al., 2002; Liu et al., 2009) and with IKK-β (Wiesner et al., 2008). In addition, α-catulin has also been shown to interact with dystrophin in the dystroglycan-dystrophin-utrophin complex, where dystroglycan mediates cell-ECM adhesion (Bozzi et al., 2009; Abraham et al., 2010; Chen et al., 2010). Knock-in of GFP into α-catulin locus allowed to show that α-catulin (GFP) is expressed by 0.02% of bone marrow hematopoietic cells, including almost all HSCs and shed new light on the cellular architecture and function of the HSC niche (Acar et al., 2015). As α-catulin may serve as a scaffold for Rho signaling, and shows structural similarities to vinculin and α-catenin, particularly in the N-terminal region, which contains binding sites for β-catenin, talin, α-actinin, and actin cytoskeleton (Kobielak and Fuchs, 2004), this suggests that α-catulin may act as a cytoskeletal scaffold protein.

Here we generated α-catulin knockout (KO) mice and used 3D epithelial cell system to study the function of α-catulin *in vivo*. α-catulin deficient mice show neural tube (NT) closure defects and are embryonically lethal. Expression of α-catulin is restricted to the lateral plate of the neural tube at the level of specific rhombomeres in the hindbrain. The neuroepithelium of α-catulin deficient mice lack apically localized actin filaments and P-Mlc2, which typically correlate with proper Rho-dependent cell constriction. To gain better inside into α-catulin function, we also used 3D model system of MDCK cells which allowed us to show that α-catulin localizes specifically to the apical membrane and is crucial for proper polarization, apical actomyosin network accumulation and distribution of active RhoA. Analysis of cell–cell contacts revealed that although adherens junction components were mostly present at the junctions of α-catulin deficient cells, they were not properly organized. Because the linkage with dynamically regulated actomyosin network is required for strong hemophilic interactions of cadherins, the loss of α-catulin might affect the integrity of apical junctions, not by influencing directly junction formation like classical α-catenin, but rather by regulating active RhoA distribution, followed by proper P-Mlc2 localization at the cytoskeleton. Our data suggest that α-catulin plays an important role during NT closure by acting as a scaffold for RhoA complex distribution, localizing activated RhoA to the apical actin-myosin network. This then results in proper spatial activation of downstream myosin to influence actin-myosin dynamics and the stability of cell-cell junctions.

## Materials and Methods

### Experimental Animals

To generate *Ctnnal1* mice, ES cell line RRJ603 containing the gene trap vector pGT2Lxf in intron 1–2 of *Ctnnal1* gene (BayGenomics) was used for blastocyst microinjection and founder breeding following standard procedures. See Supplementary Material and Methods for genotyping primers. All experiments were preapproved by The University of Southern California Institutional Animal Care and Use Committee.

### RNA Isolation and Semi-Quantitative RT-PCR

α-catulin E10.5 WT and KO embryos were collected in TRIzol (Invitrogen) and total RNA extracted using the RNeasy Micro Kit (QIAGEN). To perform semi-quantitative RT-PCR, RNA was reverse-transcribed to cDNA with SuperScript II RT (Invitrogen) using oligo dTs. For reverse-transcription, 2 μl of α-catulin 10.5E WT and KO embryo RNA was used. Primers are provided in Supplementary Material and Methods.

### Indirect Immunofluorescence Detection

α-catulin embryos were isolated in cold PBS and fixed in 4% paraformaldehyde (PFA) on ice for 30 min. Embryos were then washed well 3x in PBS. Embryos were next allowed to sink in 20% sucrose overnight at 4°C. The following day, embryos were incubated in 30% sucrose: OCT for 2 h at RT on a gentle rocker, then stored at 4°C overnight. Embryos were then embedded in OCT and sectioned at 10 μM for indirect detection of various markers. Samples were fixed in 4% PFA for 10 min and subsequently permeabilized in 0.1% Triton X-100 in PBS (PBS-T) for 10 min. Next, samples were blocked in 0.1% BSA, 2.5% HI-GS, 2.5% HI-DS in 0.1% PBS-T for 30 min at RT. Primary antibodies were diluted in 0.1% BSA in 0.1% PBS-T and incubated overnight at 4°C. Alexa Fluor 488 or 594 secondary antibodies were diluted 1:500 in blocking solution and incubated 1 h at RT. Photographs were taken using AxioImager Z1 (Zeiss). Primary antibody descriptions and dilutions are described in Supplementary Material and Methods.

### Immunohistochemistry and Scanning Electron Microscopy

Embryos were fixed in 4% PFA for 10 min, washed well in PBS, ethanol dehydrated, embedded in paraffin and sectioned 6μM thick. Samples were than deparaffinized and pretreated using antigen retrieval 2100 Retriever (Proteogenix). Endogenous peroxidase was blocked in 0.03% hydrogen peroxide for 5 min, washed in 0.3% PBS-T, blocked in 0.1% gelatin, 2.5% HI-GS, 2.5% HI-DS, 0.1% BSA in 0.3% PBS-T for 1 h and incubated with primary antibody in 0.1% BSA in 0.1% PBS-T overnight at 4°C. After washing well in 0.3% PBS-T, biotin-conjugated secondary antibodies (Vector Laboratories) were diluted 1:100 in blocking solution and incubated 1 h at RT. The ABC Kit was then used following manufacturer’s instructions (Vector Laboratories). Staining was detected using DAB Peroxidase Substrate Kit (Vector Laboratories) following manufacturer’s instructions. Scanning electron microscopy was performed in the Cell and Tissue imaging Core of USC, according to standard procedures. Antibodies are described in Supplementary Material and Methods.

### β-Galactosidase Detection by X-GAL Staining

For whole mount β-galactosidase detection, embryos were isolated in cold PBS and immediately fixed in cold 0.2% glutaraldehyde for 20 min on ice. Embryos were then washed well in cold PBS and stained in x-gal staining solution [5 mM EGTA (pH 8), 2 mM MgCl_2_, 0.2% NP-40, 0.1% sodium deoxycholate, 2 mM CaCl_2_ – before use, 5 mM potassium ferricyanide, 5 mM potassium ferrocyanide, 1 mg/mL x-gal was freshly added] overnight at 37°C with gentle shaking. Staining was stopped by washing in PBS until solution was clear. For counterstaining, embryos were sectioned 8 μM thick and then counterstained with nuclear fast red.

### Cell Line and Cell Culture Conditions

MDCK (*Madin-Darby Canine Kidney*) cell line was purchased from the American Type Culture Collection (ATCC CRL-2936). MDCK cells were cultured in *Dulbecco’s modified Eagles’s medium* (DMEM) high glucose (Biowest #L0102-500) containing 10% fetal bovine serum (FBS) (Biowest, #S181S-500) and 1% antibiotics: penicillin (100 U/ml) and streptomycin (100 μg/ml) (Biowest #L0018-100). Cell line was grown at 37°C in humidified 5% CO2/95% air atmosphere. The number of living cells was calculated by Trypan Blue staining using EVE^TM^ Automatic Cell Counter (Nano EnTek, South Korea). Cell line was regularly tested for mycoplasma contamination using a PCR-based method (Young et al., 2010).

### Silencing of α-Catulin Gene by Transfection With Small Interfering RNA and Lentiviral shRNA

MDCK cells were seeded on 6-well plate in density of 70,000 cells and the next day were transfected with specific small interfering (si)RNA targeting α-catulin gene (siCTNNAL1) (HSS112822, Thermo Fisher Scientific) or with universal negative control siRNA (siNeg) (#12935200, Thermo Fisher Scientific) using Opti-MEM medium (Thermo Fisher Scientific, #11058021) and Lipofectamine RNAiMAX Reagent (Thermo Fisher Scientific, #13778100) according to manufacturer’s instructions. Before further experiments cells were cultured for 48 h. Effectiveness of silencing of α-catulin gene was confirmed by RT-qPCR. Independent MDCK cell lines were generated using lentiviral shRNA specific to canine α-catulin (KD1 and KD2) and non-effective shRNA scrambled cassette (control). Lentiviral cassettes were designed and cloned by OriGene (pGFP-C-shLentiviral vector, TR30023 and pGFP-C-shLenti-scrambled TR30021). Lentivirus was produced using Lenti-vpak Lentiviral Packaging Kit (Origene) according to the manufacturer protocol. GFP positive cells for stable cell lines were selected by FACS sorting.

### Plasmids Transfection

To visualize active RhoA, MDCK cells were transfected with GFP-AHPH-DM (Addgene #71368) construct using Lipofectamine 3000 Transfection Reagent (Thermo Fisher Scientific #L3000001) according to manufacturer’s instructions. To visualize α-catulin protein, MDCK cells were transfected with construct of pEGFPC1 vector with cloned mouse α-catulin cDNA upstream of GFP using Lipofectamine 3000 Transfection Reagent (Thermo Fisher Scientific #L3000001) according to manufacturer’s instructions. Both constructs were verified through sequencing. Fluorescence was examined under confocal microscope LSM 700 (Zeiss) 24 h after transfection.

### RNA Isolation, cDNA Synthesis and Real-Time Quantitative PCR (RT q-PCR)

Total RNA was isolated from MDCK cell culture 2, 4, and 6 days after siRNA transfection using the RNeasy Mini Kit (Qiagen, #74106) according to manufacturer’s instructions. The concentration and purity of RNA samples were determined using DeNovix DS-11 Spectrophotometer. cDNA was synthetized from 1 μg of total RNA using the PrimeScript RT Master Mix (TAKARA BIO INC. #RR036A) in accordance with the manufacturer’s protocol. Level of expression of α-catulin gene was quantified by RT-qPCR. Each reaction mixture contained 1 μl of 25-times diluted cDNA, 6.25 μl of Fast SG qPCR Master Mix (2x) (EurX, #E0411) and 0,5 μM specific oligonucleotide primers (listed in section Supplementary Material and Methods) in a total volume of 12.5 μl. Reactions were performed in triplicates on the LightCycler 480 II (Roche) in the following conditions: 30 s at 95°C, followed by 45 amplification cycles (95°C for 10 s, 60°C for 10 s, 72°C for 10 s). Levels of expression of α-catulin gene were normalized to GAPDH. Primers are provided in Supplementary Material and Methods.

### Cyst Formation Assay

Single cell suspensions of 10,000 siRNA-treated MDCK cells were seeded on 8-well glass Millicell EZ slides (Merck Millipore, #PEZGS0816) pre-coated (37°C, 30 min) with collagen I solution (70%): matrigel (30%) (BD Matrigel Matrix, Growth Factor Reduced, BD Biosciences, #356231) mixture in the presence of 2% matrigel in culture media and cultured for 4 days. Collagen I solution was prepared as follows: 2 mg/ml collagen type I (Rat Tail High Concentration, Corning, #354249), 24 mM L-glutamine (Gibco Life Technologies, #25030-081), 20 mM HEPES (Alfa Aesar ThermoFisher, #J61047), 2.35 mg/ml Sodium Bicarbonate (Biowest, #L0680-500) and DMEM 1x.

### Polarization Analysis

Single cell suspensions of MDCK cells were seeded in density of 40,000 cells on 12 mm Nunc^TM^ Polycarbonate Cell Culture Inserts with 0,4 micron pore size in 12-well plate (ThermoFisher Scientific, #1406562) and grown for 4 days.

### Immunofluorescence Analysis

Cells for immunofluorescent staining were fixed in 4% PFA (Paraformaldehyde) for 10 min in room temperature and subsequently permeabilized in 0.1% Triton X-100 in PBS (PBS-T) for 10 min room temperature. Next, non-specific sites were blocked in 0.1% BSA, 2.5% NGS (Normal Goat Serum), 2.5% NDS (Normal Donkey Serum) in 0.1% PBS-T for 30 min at room temperature. Specific primary antibodies were diluted in 0.1% BSA in PBS-T and incubated overnight at 4°C. Secondary fluorochrome-conjugated antibodies were diluted 1:500 in blocking solution and incubated 1 h at room temperature in the dark. Primary and secondary antibodies descriptions are listed in section Supplementary Material and Methods. For actin visualization samples were incubated with phalloidin-TRITC (Fluka #77418) in dilution 1:200 for 1 h in room temperature in the dark. The cells were counterstained with nuclear dye 4’6-diamidino-2-phenylindole (DAPI; Calbiochem, # 268298) in dilution 1:1000 for 2 min in room temperature in the dark. Samples were mounted in FluorSave (#345789, Calbiochem). Images were acquired by a laser scanning confocal microscope LSM 700 (Zeiss) and analyzed with Zen2 (Blue and Black edition) Program (Zeiss). Primary antibody descriptions and dilutions are described in Supplementary Material and Methods.

## Results

### Decrease of α-Catulin Level in Mice During Development Results in Neural Tube Closure Defects

**FIGURE 1:**
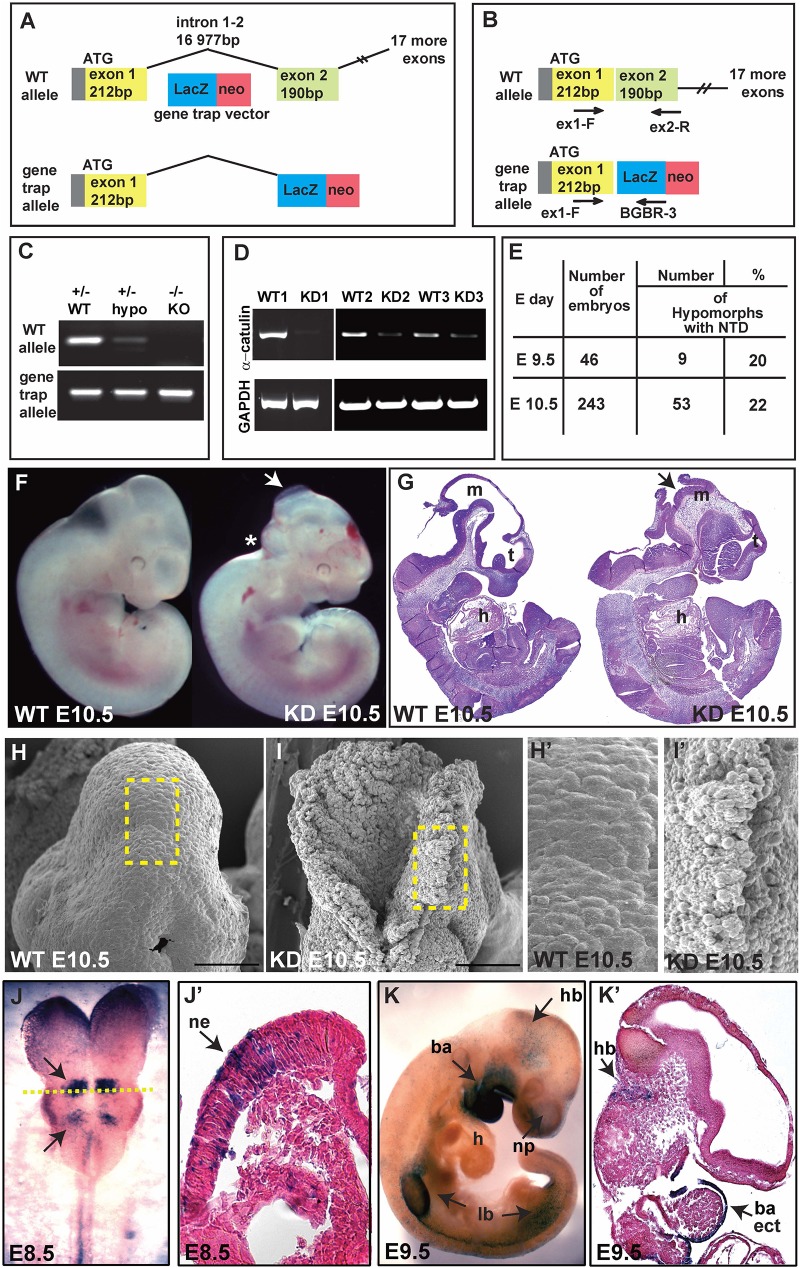
Ablation of α-catulin results in embryonic lethality at embryonic day 10.5. **(A)** α-catulin knockouts (KO) were generated by the insertion of a β-galactosidase gene trap vector into the α-catulin locus. **(B)** Genotypes of progeny were determined by using WT (ex1-F, ex2-R) and gene trap-allele-specific (ex1-F, BGBR-3) PCR primers. **(C)** Use of allele-specific primers reveals low levels of α-catulin WT allele in hypomorphs. **(D)** Hypomorphic nature of the gene trap system was confirmed by sqRT-PCR as some E10.5 KD embryos (KD2, KD3) still expressed low levels of α-catulin. **(E)** The percentage of α-catulin hypomorphic embryos manifesting neural tube closure defects. **(F,G)** Most α-catulin hypomorphic embryos die at E10.5, exhibiting neural tube closure defects, as visualized by bright field **(F)** and H&E staining **(G)** (arrows indicate open NT, asterisk marks defects in the hindbrain area). **(H,I)** Scanning electron micrographs of the hindbrain area in α-catulin WT and KD embryos at E10.5. Scale bars, 100 μm. **(H’,I’)** Magnification of the boxed areas in H and I of scanning electron micrographs of the hindbrain in α-catulin WT and KD embryos at E10.5. **(J–K’)** Utilizing the β-galactosidase reporter, we can visualize α-catulin expression pattern at various stages by x-gal staining. **(J,J’)** At E8.5, α-catulin is expressed in the neuroepithelium, particularly in the hindbrain region and along the neural tube as indicated by arrows. The cross-section in **(J’)** is indicted by the dash line in **(J)**. **(K,K’)** At E9.5 α-catulin is expressed in the hindbrain, the ectoderm of the branchial arches and nasal pits, and in the limb buds as indicated by arrows. m-mesencephalon, t-telencephalon, h-heart, hb-hindbrain, ne-neuroepithelium, ba-branchial arches, lb-limb bud, np-nasal pit, ect-ectodermal.

To gain insight into the physiological function of α-catulin, we generated straight α-catulin knockout mice using embryonic stem (ES) cells with a gene trap vector, where β-galactosidase is inserted into the α-catulin locus (Bay-Genomic cell line RRJ603) (Figure 1A). We confirmed the correct insertion of the trapped vector in the α-catulin locus by sequencing cDNA isolated from ES cells (Supplementary Figure S1E) and genotyping (Figures 1B,C). α-catulin knockout mice die very early embryonically at day E10.5, with different severity of phenotype, which is likely due to the hypomorphic nature of the gene trap, as confirmed by sqRT-PCR using RNA isolated from the embryos (Figure 1D). However, those animals consistently show defects in NT closure (Figures 1F,G and as summarized in Figure 1E). Littermates lacking α-catulin exhibit massive malformation of the developing brain. The first obvious phenotype, a failure in neural tube closure, is clearly visible already at E10.5. In mice, the neural tube closure is initiated at the hindbrain/cervical boundary, at the midbrain/forebrain boundary, and at the most anterior part of the telencephalon from where closure spreads into the remaining areas of the neural tube (Nikolopoulou et al., 2017). Based on our observations, we suggest that α-catulin embryos might have a failure of the initiation point at the hindbrain/cervical boundary closure. NT closure defect is accompanied by a general increase in deformability at the tissue-level, especially in the hindbrain area (asterisk in Figure 1F). Loosely organized pattern of cells in open neural tube in the hindbrain area of α-catulin knockdown (KD) mice as compared to wild type (WT) littermate is observed in scanning electron microscopic images (Figures 1H,I,H’,I’). Utilizing the β-galactosidase gene trap vector, α-catulin expression can be studied by staining with x-gal since heterozygous α-catulin ± mice appeared normal. Surprisingly, the pattern of expression of α-catulin at early stages of the mouse development was very dynamic. Staining for x-gal, at E8.5, α-catulin is expressed in the neuroepithelium, at the level of specific rhombomeres in the hindbrain and along the neural tube as indicated by arrows (Figures 1J,J’). At E9.5, α-catulin is expressed in the hindbrain, the ectoderm of the branchial arches and nasal pits, as well as in the limb buds as indicated by arrows (Figures 1K,K’). At E10.5, α-catulin expression is also visible in the dermomyotome, developing eye, nasal pits and limb buds (Supplementary Figures S1A,A’,A”). Around day E14.5 α-catulin expression appears strongly around the eye, otic vesicle (arrows in Supplementary Figure S1B) and in the dorsal root ganglia (arrows in Supplementary Figures S1B,B’) where it persists after the birth (arrows in Supplementary Figures S1C,C’). In newborn (NB) mice, α-catulin is also expressed in the neural crest derived innervation (arrows in Supplementary Figure S1D). Taken together, this data reveal, for the first time, a crucial role for α-catulin during mouse NT closure, and highlights the dynamic expression pattern of α-catulin that can correspond to its importance in the plasticity of epithelial tissues during morphogenesis.

### Neuroepithelium of α-Catulin Deficient Embryos Lacks Apically Localized Actin Filaments and P-Mlc2

**FIGURE 2:**
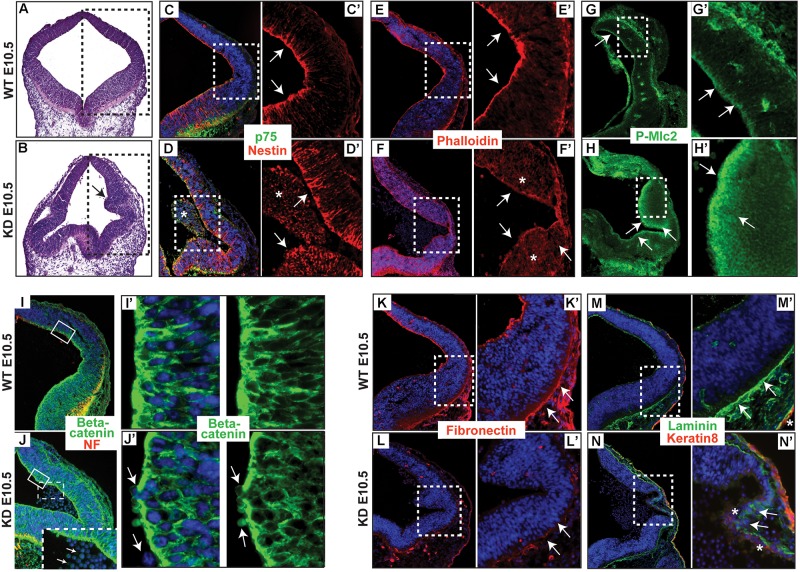
α-catulin hypomorphic mice lack apically localized actin filaments and show disorganization of integrin-mediated basement membrane assembly. **(A,B)** Coronal sections of α-catulin WT (top panel) and hypomorphic (bottom panel) mice at the level of hindbrain area were stained with H&E to visualize defects in the neuroepithelium of α-catulin KD. Serial sections of boxed areas in **(A,B)** were immunostained with specific antibodies as indicated. **(C,C’,D,D’)** α-catulin WT and KD E10.5 embryos were stained with a neuronal keratin filament nestin and p75 neurotrophin receptor **(E,E’,F,F’)** as well as phalloidin, for actin filaments. **(G,G’,H,H’)** Phospho-specific antibody against myosin light chain 2 (P-Mlc2). Arrows in **(C’,E’,G’)** indicate apical accumulation of specific markers in WT neuroepithelium, whereas arrows in **(D’,F’,H’)** indicate diffuse distribution of those markers in α-catulin KD. Asterisks in **(D,D’)** mark masses of cells separated from the neuropeithelium. Asterisks in **(F’)** mark diffuse distribution of actin. **(I,I’,J,J’)** Neuroepithelium of WT and α-catulin KD embryos were co-stained with neurofilament (NF) and β-catenin to visualize adherens junctions. **(I’,J’)** enlarged boxes of β-catenin staining. Arrows in **(J’)** indicate loosely connected, separating cells. Dashed box in **(J)** is enlarged at the bottom to indicate by arrows separating cells. **(K–N’)** Integrin-mediated basement membrane (BM) assembly is affected in the neuroepithelium of KD mice, as examined by components of the extracellular matrix, fibronectin **(K–L’)** and laminin **(M–N’**). α-catulin WT and KD E10.5 embryos stained with BM markers as indicated. **(K,K’,L,L’)** Staining with fibronectin, ECM component of basement membrane. **(M,M’,N,N’)** Staining with laminin, ECM component of basement membrane, co-stained with keratin 8, epidermal layer marker. Arrows in **(K’,M’)** indicate properly formed BM, whereas arrows in **(L’,N’)** indicate lack of BM formation. Asterisks in **(N’)** indicate misslocalized expression of keratin 8, whereas asterisk in **(M’)** marks properly expressed keratin 8 in the developing epidermis. Boxed region is magnified in each following frame **(C’,D’, E’,F’,G’,H’,I’,J’,K’,L’,M’,N’)**. The antibodies used are indicated and color-coded according to secondary antibodies, blue indicates nuclear staining.

Actin and many of its associated proteins accompanied by apical accumulation of Rho and contractile actomyosin network, regulating apical constriction, were shown to play an important role in neurulation (Kinoshita et al., 2008). To determine the distribution of actin and keratin filaments in the neuroepithelium of α-catulin deficient mice, we stained coronal sections with phalloidin, nestin and p75 neurotrophin receptor specific antibodies. The neuroepithelium of α-catulin deficient mice showed lack of apical accumulation of actin (Figures 2E,E’,F,F’) and nestin filaments (Figures 2C,C’,D,D’). As seen by hematoxylin and eosin (H&E) staining, neuroepithelium of α-catulin deficient mice showed extra bending (arrow in Figure 2B) as compared to WT littermates (Figure 2A). As it was described previously, that α-catulin can act as a scaffold for Rho complex distribution (Park et al., 2002), we wanted to check if this observation is also relevant *in vivo* by staining hindbrain sections of α-catulin E10.5 WT and KD embryos with antibody for downstream target of active RhoA – P-Mlc2. The lack of apical accumulation of cytoskeleton and defects in neural tube bending and closure correlated with the lack of proper apical localization of P-Mlc2 in α-catulin-deficient neuroepithelium. P-Mlc2 appeared as a diffused signal extending through the broad area of α-catulin-deficient neuroepithelium (arrows in Figures 2H,H’) in comparison to WT (arrows in Figures 2G,G’). Since abnormal actomyosin accumulation might also affect the structure and/or function of apical junctional complexes, which are implicated in neuroepithelial morphogenesis (Nishimura and Takeichi, 2009) we also checked the localization of cell-cell junction component, β-catenin in those embryos. Immunostaining revealed proper localization of β-catenin, specifically localized to the lateral membranes of neuroepithelial cells with tight cell-cell junctions (Figures 2I,I’) in WT embryos. In contrast, α-catulin deficient embryos, despite strong β-catenin staining at the membranes, exhibited a slightly disorganized cell-cell junctions with less elongated cells and more rounded nuclei, suggesting defects in polarization of the neuroepithelium (Figures 2J,J’). In addition, some the cells were loosely separating from the neuroepithelium of α-catulin deficient embryos (arrows in Figures 2J,J’). This phenotype was quite common as it is also noticeable in neuroepithelium of α-catulin deficient embryos in Figures 2D’,N’ (asterisks), indicating instability of cell-cell junctions. High level of tissue disorganization in α-catulin-deficient mice also affected integrin-mediated basement membrane assembly of the neuroepithelium, as judged by the staining of extracellular matrix components, fibronectin and laminin, which were weakly expressed in α-catulin-deficient mice (Figures 2L,N, arrows in Figures 2L’,N’) as compared to WT littermates (Figures 2K,M, arrows in Figures 2K’,M’). Surprisingly, in some areas non-neuronal keratin 8 staining was observed in α-catulin-deficient mice thinner layer of neuroepithelium (Figure 2N, asterisks in Figure 2N’) which in WT littermates is only present in developing epidermis (Figure 2M, asterisk in Figure 2M’). Our initial observations of neuroepithelium architecture in α-catulin-deficient mice suggest that lack of proper apical actin accumulation with P-Mlc2 distribution might be essential in enabling apical constriction and adherens junctions remodeling as the neuroepithelium undergoes morphogenesis.

### Disorganization of Neuroepithelium in α-Catulin-Deficient Embryos Does Not Affect Differentiation Program of the Neuroprogenitors

**FIGURE 3:**
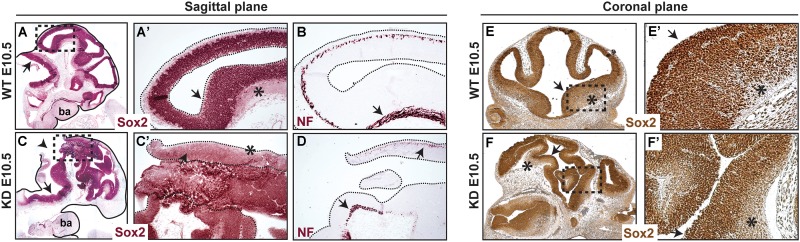
Abnormal morphology of neuroepithelium in α-catulin hypomorphic embryos. Sections of α-catulin KD (lower panel) and wild-type E10.5 embryos (upper panel) were stained with Sox2 (neural progenitors) and neurofilament (NF) (early neuronal differentiation marker) for comparison. Sections show high level of disorganization, with extra bending of the neuroepithelial layers, in α-catulin KDs. Immunohistochemical stainings show some degree of localized Sox2 and NF expression in α-catulin KDs, compared to WT embryos. **(A–D)** Sagittal plane of the embryos, **(E,F)** coronal plane of the embryos. Arrows in **(A,A’,B,C,C’,D,E,E’,F,F’)** show localized expression of the indicated markers, arrowhead in **(C)** show the opening of the neural tube, asterisks indicate NF positive differentiating cells. Magnified regions **(A’,C’,E’,F’**) are as indicated **(A,C,E,F)**. ba, branchial arches.

Since neural tube closure defects were consistent and predominant in α-catulin-deficient mice, we focused further analyses on the neuroepithelium. The neuroepithelium of normal E10.5 embryos consist of the ventricular zone (VZ) layer, containing proliferating neural precursors which express Sox2, and the mantle containing post-mitotic neurons which start to express neurofilament (NF). α-catulin-deficient mice show some degree of localized expression of Sox2 (arrows in Figures 3C,C’,F,F’) as compared to its wild type (WT) littermate (arrows in Figures 3A,A’,E,E’). Post-mitotic neurons which start to express neurofilament are also visible in α-catulin-deficient neuroepithelium (arrows in Figure 3D) as compared to WT littermates (arrow in Figure 3B). Despite high level of disorganization, which is even more obvious in the coronal plane section stained with Sox2 (Figure 3F), as compared to WT (Figure 3E), Sox2 staining still localizes in the ventricular layer with decreased expression in mantle layer (asterisks in Figures 3E,E’,F,F’).

### Alpha-Catulin Depletion in MDCK Cells Results in Altered Cyst Formation

**FIGURE 4:**
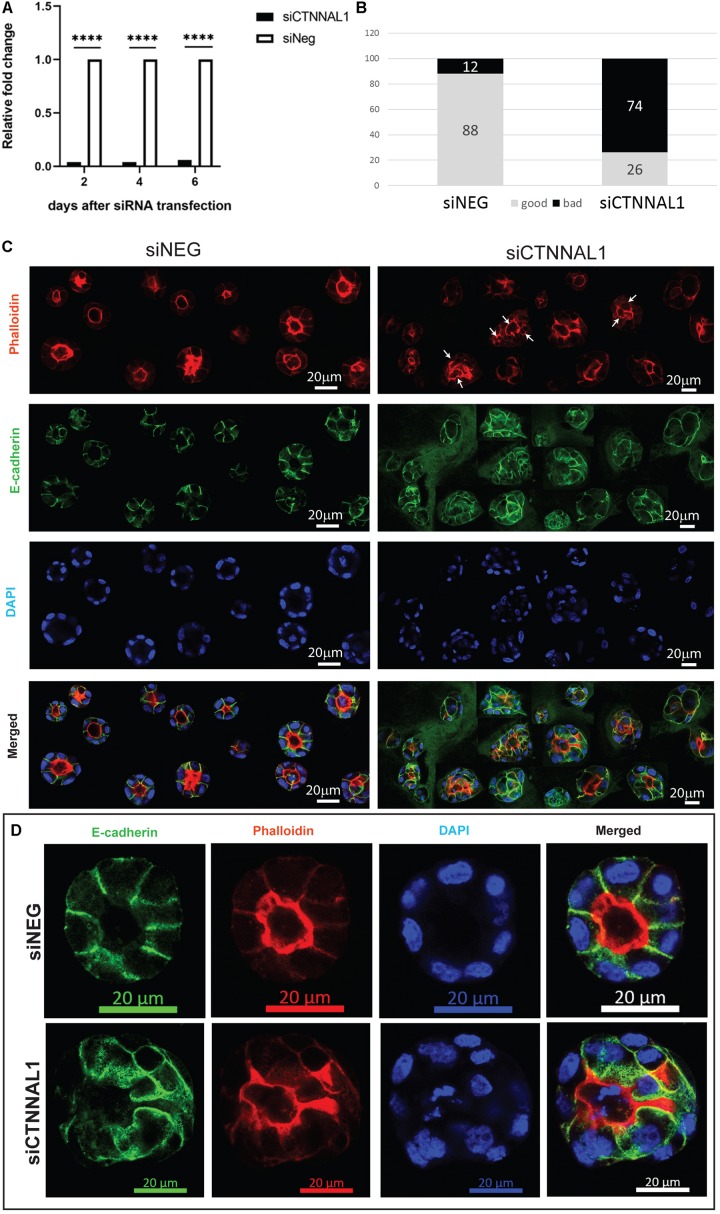
Effect of silencing of α-catulin on cyst formation by MDCK cells. **(A)** RT-qPCR analysis of α-catulin level in MDCK cells after 2, 4, and 6 days after transfection with siRNA specific to α-catulin (siCTNNAL1) or with negative siRNA (siNEG) as a control. The results were normalized to GAPDH levels. The level of α-catulin in siCTNNAL1 MDCK cells was at least 95% lower than in control (siNEG) in all cases, what indicates efficient knock-down of α-catulin gene. **(B)** Quantification of properly and improperly formed cysts by MDCK cells transfected with siRNA specific to α-catulin (siCTNNAL1) or with negative siRNA (siNEG). MDCK cells were transfected with siCTNNAL1 and siNEG siRNA and 48 h after transfection, single cell suspension of MDCK cells was seeded in collagen/matrigel mixture. After 4 days of culture, cysts that formed from single cells, were fixed and immunostained with specific antibody against E-cadherin, stained with phalloidin and counterstained with DAPI and subjected to confocal microscopy analysis. Whole wells with cyst embedded in collagen/matrigel mixture were scanned under 40x magnification and cysts consisting of 5 or more cells were divided in two types and quantified. First type named as “good” cysts was containing regular, single layer, single lumen, circular, properly formed cysts. Second type of cyst named as “bad” contained all cysts that were not properly formed, did not have a lumen or had multiples lumens, were not circular or cells in the cyst have not formed single layer. All these cyst types were quantified in both groups (siCTNNAL1 and siNEG) and the results are presented as percentage contribution of each cyst. **(C)** View of cysts appearance formed by siNEG and siCTNNAL1 MDCK cells immunostained with E-cadherin antibody (arrows indicate multiple lumens in cysts). For more detailed examination, randomly chosen cysts formed by siNEG and siCTNNAL1 MDCK cells were imaged under 63x magnification. Presented figure was created as a result of connection of different cyst images. **(D)** Representative images of cysts formed by siNEG and siCTNNAL1 MDCK cells immunostained with E-cadherin antibody imaged under 63x magnification. Scale bar 20 μm.

**FIGURE 5:**
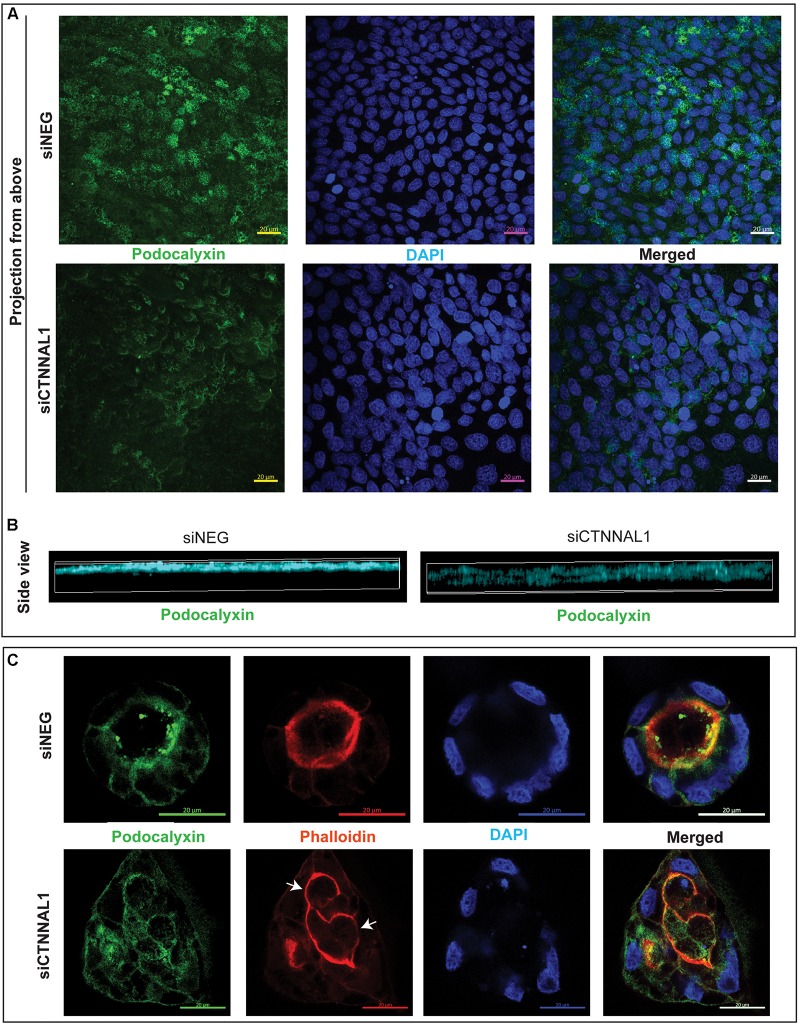
Effect of silencing of α-catulin on localization of polarity marker, podocalyxin in MDCK cells. **(A)** Immunofluorescence staining of podocalyxin in MDCK cells grown on Transwell Nunc filters. MDCK cells, seeded the day before, were transfected with siRNA specific to α-catulin (siCTNNAL1) or with negative siRNA (siNEG) as a control and after 48 h were trypsynized, counted and seeded in density of 40,000 cells on 12 mm Transwell Nunc filters and grown for 4 days. Then, cells were stained with a podocalyxin specific antibody. Images present confocal microscopy analysis in xy axis of cell monolayer (Projection from above) that revealed decreased amount of podocalyxin protein in siCTNNAL1 MDCK cells. Scale bar 20 μm. **(B)** 3D visualization of orthogonal views in xz axis show decreased and dispersed localization of podocalyxin in siCTNNAL1 MDCK cells in comparison to highly specific apical localization of podocalyxin in control cells (siNEG). **(C)** Representative cysts formed by siCTNNAL1 and siNEG MDCK cells immunostained with antibody specific to podocalyxin. Podocalyxin shows mainly apical localization in cysts formed by siNEG MDCK cells, indicating proper polarization of the cells. In contrast, in cysts formed by siCTNNAL1 MDCK cells, we could not observe such a specific localization of this protein, which suggests inappropriate cells polarization (arrows indicate multiple lumens in the cyst). Images present single confocal section taken through the middle of a spherical cyst. Scale bar 20 μm.

The subcellular organization and dynamics of actomyosin network are challenging to characterize in developing embryos. Therefore, to get better insight into the role of α-catulin in the actomyosin coordination in epithelial cells, in parallel to our study on mice, we used MDCK canine kidney epithelial cells, that are widely used for studying cell polarity, actin organization and adhesion. The effect of suppression of α-catulin on the organization of epithelial cells in 3D organotypic culture was assessed in cysts formed from MDCK II cells transfected with siRNA targeting α-catulin gene (siCTNNAL1) and control (siNEG). The efficiency and stability of knockdown was verified by RT-qPCR, on days 2, 4, and 6 after siRNA transfection. The level of α-catulin in siCTNNAL1 MDCK cells was at least 95% lower in siCTNNAL1 than in control (siNEG) cells at all time points (Figure 4A). Deficiency of α-catulin resulted in impaired cyst formation (Figure 4C) with high level of cells disorganization, often forming multiple lumens (arrows in Figures 4C, 5C) or no visible lumens at all. To perform quantification, we counted cysts in both groups (siCTNNAL1 and siNEG) that contained 5 or more cells, naming “good,” cysts that contained regular, single layer and single lumen and “bad,” cysts with irregular or multiple layers, without lumen or with multiple lumens. In control siNEG cells “good” cysts constituted 88% and “bad” cysts only 12%, whereas in siCTNNAL1 cells proportion between “good” and “bad” cysts were 26% and 74% respectively (*n* = 100 for each group) (Figure 4B and Supplementary Figures S2A,B). Cell-cell contacts were assessed by the staining with E-cadherin specific antibody. In control cells, E-cadherin signal was present essentially at cell–cell contacts of cysts with a spherical monolayer (Figures 4C,D). After depletion of α-catulin, E-cadherin was still present at cell-cell contacts, however, staining was diffused in some areas and disorganized (Figure 4D). We further analyzed polarity status in these MDCK cysts by staining actin to visualize the apical domain. As expected, control MDCK cysts formed a central single lumen represented with Phalloidin staining, whereas α-catulin depleted MDCK cells formed multilumen cysts with less abundant apical actin that partially spread to the lateral domains (Figure 4D). Consistent data were obtained using independent shRNA stably expressed in MDCK cells using lentiviral transduction (Supplementary Figures S3, S4). To further investigate possible disruption in polarization in cysts formed by α-catulin deficient MDCK cells, we immunostained them with antibody specific for well-established apical marker, podocalyxin. We noticed proper apical accumulation of podocalyxin in cysts formed by control (siNEG) cells and limited accumulation of this protein in cysts formed by siCTNNAL1 MDCK cells (Figure 5C). In addition, MDCK cells transfected with siRNA targeting α-catulin gene (siCTNNAL1) and control (siNEG) were seeded on filters and 4 days after transfection podocalyxin was visualized localizing on the apical membrane of control (siNEG) monolayers (Figure 5A upper panel and Figure 5B left image). Removal of α-catulin resulted in diffuse localization of podocalyxin and lack of proper apical accumulation of this marker, visible in projection from above and in the side view (Figure 5A lower panel, Figure 5B right image). Consistent data were obtained using independent shRNA stably expressed in MDCK cells using lentiviral transduction (Supplementary Figure S5B). These findings indicate that depletion of α-catulin has a strong effect on cell polarity, with high number of cysts presenting multilumens. This is also in line with our observations of the neuroepithelium of α-catulin deficient mice that lacked apical accumulation of actin and despite β-catenin staining at the membranes, exhibited a markedly disorganized cell-cell junctions with some cells loosely separating from the neuroepithelium.

### Alpha-Catulin Is Important for Proper Spatial Distribution of Active RhoA in Polarized MDCK Cells

**FIGURE 6:**
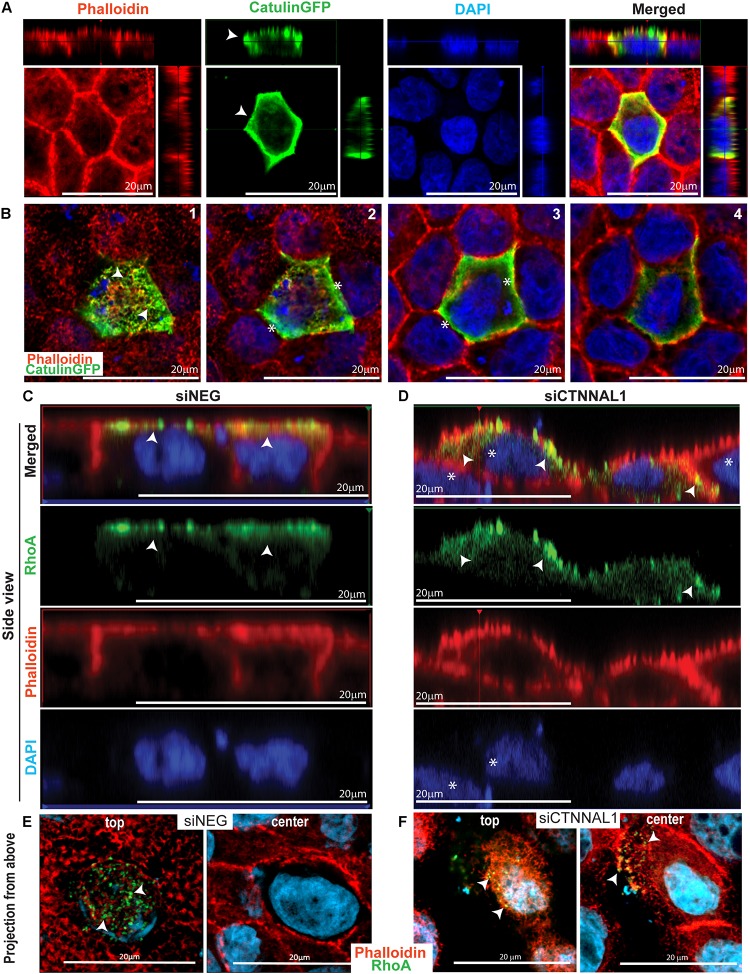
α-catulin dependent localization of active RhoA in polarized MDCK cells. **(A)** Localization of α-catulin in polarized MDCK cells. MDCK cells were seeded in density of 40,000 cells on 12 mm Transwell Nunc filters and grown for 3 days, then they were transfected with α-catulin GFP fusion plasmid construct and grown for additional 1 day. After that, cells were stained with a primary anti GFP antibody as well as with phalloidin to visualize actin. Immunofluorescence staining visualized by xy analysis of cell monolayer by confocal microscopy revealed strong membrane localization of α-catulin (green) in polarized MDCK cells. Orthogonal views (projections) of polarized MDCK cells presented a clear apical and lateral localization of α-catulin in filter-grown polarized MDCK cells (arrowheads), scale bar 20 μm. **(B)** Confocal microscopy analysis of filter-grown MDCK cells transfected with α-catulinGFP phusion plasmid construct in XYZ axis. Subsequent photos (1–4) are showing visualization of different Z positions from most apical (1) to basal (4). We could observe apical localization of a-catulin protein (arrowheads in picture 1) as well as its presence on the cell membrane (asterisks in picture 2–3), scale bar 20 μm. **(C,D)** Localization of active RhoA protein in polarized siNEG **(C)** and siCTNNAL1 **(D)** MDCK cells in side view. MDCK cells were seeded in density of 40,000 cells on 12 mm Transwell Nunc filters and grown for 3 days, then they were transfected with active RhoAGFP reporter and grown for additional 1 day. After that, cells were stained with phalloidin to visualize actin. Immunofluorescence staining visualized by xz analysis of cell monolayer by confocal microscopy revealed apical membrane localization of active RhoA protein (arrowheads) and phalloidin staining showed nicely formed monolayer and cell-cell contacts in polarized siNEG MDCK cells. In contrast, in monolayer formed by siCTNNAL1 MDCK cells, active RhoA was present also in lateral membrane (arrowheads) and cells often were overlapping on each other (asterisks), scale bar 20 μm. **(E,F)** Localization of active RhoA protein in polarized siNEG **(E)** and siCTNNAL1 **(F)** MDCK cells in projection from above. (**E,F** top) Apical surface of control siNEG (**E** top) and siCTNNAL1 (**F** top) MDCK cells. We observed highly specific signal from active RhoA protein in control cells in their apical surface and some signal from this protein in siCTNNAL1 cells but it was weaker and less organized. (**E,F** center) Cross section from nucleus plane of control siNEG (**E** center) and siCTNNAL1 (**F** center) MDCK cells. In this plane there was no active RhoA in control cells, whereas in siCTNNAL1 we could observe signal indicating presence of this protein (arrowheads).

It was previously shown that apical accumulation of Rho in developing chick embryos undergoing folding of the neural plate during neural tube formation correlated with similar accumulation of activated myosin II (Kinoshita et al., 2008). The timing of accumulation and biochemical activation of both RhoA and myosin II coincided with the dynamics of neural tube formation. As our observation in mice and 3D cultures strongly suggested that α-catulin is an important mediator of apical accumulation of actin and signaling to actin/myosin cytoskeleton, we wanted to check if α-catulin could be acting as a scaffold for RhoA distribution. This could be potentially true since α-catulin was previously described as a scaffold for Lbc-RhoGEF affecting RhoA signaling (Park et al., 2002). First we tested intercellular localization of α-catulin in polarized cells. We used MDCK cells grown for 3 days on collagen-coated glasses, transfected with α-catulin-GFP expressing plasmid (efficiency of transfection shown in Supplementary Figure S6B), previously verified by Western Blot (Supplementary Figure S6A) since canine specific α-catulin antibody was not available. Cells were grown for additional day, followed by staining with anti-GFP antibody and phalloidin to visualize actin. Analysis of immunofluorescent staining of cell monolayers by confocal microscopy revealed strong co-localization of α-catulin and actin at the apical membrane spanning till the cell-cell junctions (arrowheads in Figure 6A). Z-stack analysis of the apical membrane clearly demonstrated co-localization of α-catulin with apical actin (arrowheads in Figure 6B-1) and some enrichment at the cell-cell junctions (asterisks in Figures 6B-2,B-3). Because apical localization and activity of RhoA is a central feature of establishing efficient contractile actinmyosin network, we next investigated if depletion of α-catulin would affect this activity. In order to analyze the distribution of active RhoA in α-catulin depleted polarized MDCK cells, 2 days after transfection with siRNA targeting α-catulin gene (siCTNNAL1) and control (siNEG), cells were trypsynized, counted and seeded on filters and grown for 3 days, then they were transfected with previously described GFP-AHPH sensor known to bind only to active RhoA (RhoA-GTP) and grown for additional day (Tse et al., 2012; Priya et al., 2015). After that, cells were stained with phalloidin to visualize actin. We examined GFP signal that indicated active RhoA localization in polarized α-catulin deficient and control MDCK cells and noticed specifically localized active RhoA on apical membrane in control cells (arrowheads in Figure 6C) and its random distribution (present also in basolateral membrane) in α-catulin depleted MDCK cells (arrowheads in Figure 6D). Z-stack analysis of the apical membranes of α-catulin control and deficient cells strongly demonstrated exclusive apical accumulation of active RhoA in control cells and random distribution throughout the α-catulin deficient cell (arrowheads in Figures 6E,F). Our data suggest that α-catulin might be a scaffold for active RhoA localization to the cell apex.

### Depletion of α-Catulin in MDCK Cells Results in Disorganization of Polarized Monolayer, However, Adherens Junction Components Are Still Present at the Cell-Cell Contacts

**FIGURE 7:**
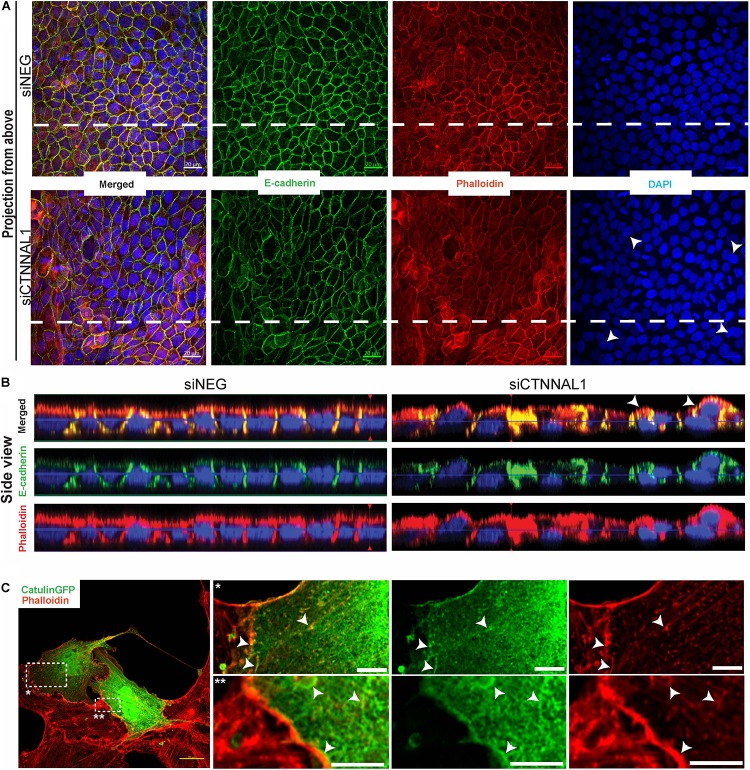
Organization of polarized monolayer of MDCK cells after α-catulin depletion. **(A)** Immunofluorescence staining of E-cadherin in MDCK cells grown on Transwell Nunc filters. MDCK cells, seeded the day before, were transfected with siRNA specific to α-catulin (siCTNNAL1) or with negative siRNA (siNEG) as a control and after 48 h were trypsynized, counted and seeded in density of 40,000 cells on 12 mm Transwell Nunc filters and grown for 4 days. Then, cells were stained with a primary anti- E-cadherin antibody. Images present confocal microscopy analysis in xy axis of cell monolayer (Projection from above). **(B)** Side view of filter-grown MDCK cells transfected with siRNA specific to α-catulin (siCTNNAL1) or with negative siRNA (siNEG) immunofluorescence stained with E-cadherin. Arrowheads in **(A,B)** indicate cells overlapping each other. Dashed lines in **(A)** indicate side view presented in **(B)**. **(C)** Partial co-localization of α-catulin with actin (phalloidin) in MDCK cells. MDCK cells were seeded on collagen-coated glasses and the next day cells were transfected with α-catulinGFP fusion plasmid construct. After 24 h post-transfection cells were stained with a primary anti GFP antibody as well as with phalloidin to visualize actin. As a result, staining confirmed partial co-localization of α-catulin (green) with actin (red) on the cell membrane and in the actin cytoskeleton (white arrowheads). Boxed areas are enlarged. Scale bars: main photo on the left side 20 μm, enlarged boxes 5 μm.

**FIGURE 8:**
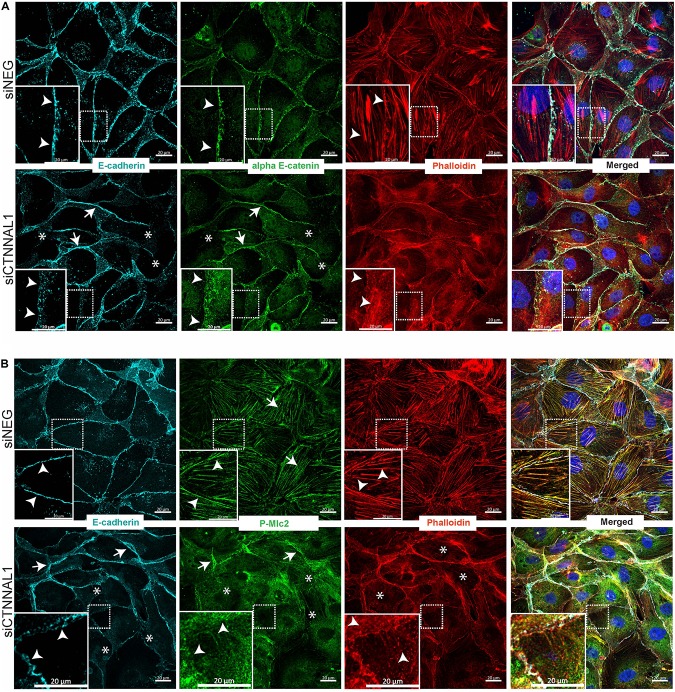
Effect of silencing of α-catulin on actin cytoskeleton organization as well as on distribution and localization of E-cadherin, αE-catenin and phosphorylated myosin in MDCK cells. **(A)** Immunofluorescence staining of E-cadherin, αE-catenin and phalloidin in MDCK cells grown on collagen-coated glasses. MDCK cells were seeded in density of 10,000 cells per well of 24 well plate on collagen-coated glasses and the next day they were transfected with siRNA specific to α-catulin (siCTNNAL1) or with negative siRNA (siNEG) as a control. Forty eight hours after transfection cells were fixed and stained with E-cadherin, αE-catenin and phalloidin and counterstained with DAPI and then were subjected to confocal microscopy analysis. Actin cytoskeleton in control cells is well-arranged, with clearly visible actin fibers along the cells (arrowheads upper panel), whereas in siCTNNAL1 cells actin cytoskeleton is significantly less organized, with very thin actin fibers (arrowheads lower panel). αE-catenin and E-cadherin show unequal distribution in siCTNNAL1 samples (arrowheads mark dispersed, arrows mark enriched distribution and asterisks mark absence of the staining) in comparison to sharp localization of these proteins on the cell membrane of control cells (siNEG) (arrowheads). Boxed areas are enlarged. Scale bar 20 μm. **(B)** Immunofluorescence staining of E-cadherin, phospho myosin 2 and phalloidin in MDCK cells grown on collagen-coated glasses. MDCK cells were seeded in density of 10,000 cells per well of 24 well plate on collagen-coated glasses and the next day they were transfected with siRNA specific to α-catulin (siCTNNAL1) or with negative siRNA (siNEG) as a control. 48 h after transfection cells were fixed and stained with E-cadherin, P-Mlc2 (phospho myosin light chain 2) and phalloidin and counterstained with DAPI and then were subjected to confocal microscopy analysis. In addition to previous observations about appearance of actin cytoskeleton and E-cadherin distribution, we noticed profound changes in distribution of P-Mlc2 in siCTNNAL1 cells (arrows mark strong accumulation of P-Mlc2 at the membranes, arrowheads mark diffused presence at the membranes and asterisks indicate diffused pattern of P-Mlc2 throughout the cell body) in comparison to control cells (siNEG) (arrowheads mark specific staining at the membranes, arrows indicate actomyosin cables). Boxed areas are enlarged. Scale bar 20 μm.

Since polarization of epithelial cells and distribution of apical proteins is dependent on proper cell-cell junction formation and stabilization, we wanted to get better insight into the organization of adherens junction in α-catulin deficient cells. MDCK cells transfected with siRNA targeting α-catulin gene (siCTNNAL1) and control (siNEG) were seeded on filters for 4 days, followed by immunostaining with antibody specific for E-cadherin and stained with phalloidin to visualize actin. Confocal microscopy analysis in xy axis (projection from above) and xz (side view) of stained cells monolayer revealed disrupted formation of monolayer in α-catulin deficient cells, despite preserved cell-cell contacts as judged by E-cadherin staining (Figures 7A,B). In control, MDCK cells formed single-cell monolayer, with equal cell sizes and even, flat apical surface of monolayer. In contrast, α-catulin deficient MDCK cells created irregular, “bumpy” layer, with cells often overlapping each other (arrowheads in Figure 7A lower panel, Figure 7B right panel and asterisks in Figure 6D), indicating lack of stabilization in cell-cell contacts. Consistent data were obtained using independent shRNA stably expressed in MDCK cells (Supplementary Figure S5A). In order to examine an intercellular localization of α-catulin, we used MDCK cells grown on collagen-coated glasses, transfected with α-catulin-GFP expressing plasmid (previously verified by Western Blot Supplementary Figure S6A) followed by phalloidin staining to visualize actin. α-catulin-GFP was enriched at the cell-cell junctions (Figure 7C and arrowheads in enlarged boxes marked by double star). However, more pronounced localization was observed along the filamentous actin (Figure 7C and arrowheads in enlarged boxes marked by single star). Accumulation of α-catulin-GFP was not so dramatic as other typical cell-cell junction proteins, e.g., αE-catenin. We next assessed the distribution of adherens junction components in flat cultures of α-catulin deficient and control cells. Co-staining of E-cadherin and αE-catenin revealed specific localization of both proteins to adherens junctions in control MDCK cells, showing equal distribution of junctional complexes between cells (arrowheads in Figure 8A upper panel). Actin cytoskeleton was properly organized in control cells, with enrichment at the membrane and actin cables spanning throughout the cells (arrowheads in Figure 8A upper panel). In the absence of α-catulin, we observed unequal distribution of E-cadherin and αE-catenin with some areas enriched with those proteins at the junctions (arrows in Figure 8A lower panel), some with slightly diffused pattern near the membrane (arrowheads in Figure 8A lower panel) and some lacking the staining at all (asterisks in Figure 8A lower panel). Actin cytoskeleton in α-catulin deficient MDCK cells was not properly organized with limited accumulation at the junctions as an actin belt, that would indicate proper junctions stabilization (arrowheads in Figure 8A lower panel). Analysis of active P-Mlc2, downstream target of RhoA, indicated that depletion of α-catulin in MDCK cells resulted in lack of proper localization of active P-Mlc2, which could explain defects in the stability of adherens junctions. In the α-catulin deficient cells, P-Mlc2 staining was strong predominantly in the areas where cell-cell junctions were formed, as indicated by E-cadherin localization (arrows in Figure 8B lower panel). In some areas, P-Mlc2 staining was slightly diffused at the membranes which also correlated well with E-cadherin staining (arrowheads in Figure 8B lower panel). Most of the staining appeared as a diffused pattern throughout the cells without noticeable actomyosin cables (asterisks in Figure 8B lower panel). In contrast, control cells showed well organized P-Mlc2 staining, enriched at the junctions (arrowheads in Figure 8B upper panel) and forming actomyosin network of cables (arrows in Figure 8B upper panel). Analysis of cell–cell contacts revealed, that although adherens junction components were mostly present at the junctions of α-catulin deficient cells; however, they were not properly organized, which is consistent with the observation in mouse embryos (Figures 2I,J). Because the linkage with dynamically regulated actomyosin network is required for strong hemophilic interactions of cadherins, the loss of α-catulin might affect the integrity of apical junctions. It is important to note that the lateral membrane contacts are still formed in α-catulin depleted cells although not stabilized. This indicated that the loss of α-catulin might affect the integrity of apical junctions not by influencing junction formation directly, but rather by regulating active RhoA distribution, followed by proper P-Mlc2 localization at the cytoskeleton. Importantly, the lack of apical accumulation of cytoskeleton and defects in neural tube bending and closure correlated with the lack of apically localized P-Mlc2 in α-catulin-deficient neuroepithelium (Figures 2H,H’) as compared to the WT littermate neuroepithelium (Figures 2G,G’).

## Discussion

Actin and myosin II play a central role in the morphogenetic movements. The actomyosin cytoskeleton generates contractile forces in individual cells that can be transmitted between cells of a tissue through cell–cell junctions coordinating global changes in tissue organization. Although the importance of actin, myosin II, RhoA and cell-cell junctions has been clearly demonstrated in those processes, the exact mechanisms by which actomyosin networks drive cell shape changes and movements are still poorly understood (Priya and Yap, 2015; Nikolopoulou et al., 2017). In the present study, we identified the critical role of α-catulin in neurulation during mouse embryonic development. The neuroepithelium of α-catulin-deficient mice lack apically localized actin filaments and show diffused P-Mlc2 staining, which typically correlate with proper Rho-dependent cell constriction. Our studies using well-defined 3D model system of MDCK cells allowed us to show that α-catulin localizes specifically to the apical membrane and the apically-localized α-catulin is crucial for proper polarization, actomyosin network organization, and distribution of active RhoA. Function of α-catulin as a scaffold for active RhoA, actin, and myosin might cause tension generation that not only results in the apical constriction thereby bending the neural plate, but also influence cell-cell junction stability since lateral membrane contacts are still formed in α-catulin depleted cells, but not stabilized.

Despite severe defects in the organization of F-actin, we find that proteins of adherens junctions complexes localize to cell-cell membranes in α-catulin depleted neuroepithelium of mouse embryos and MDCK cells. However, the cell-cell membranes are not stable since the cells are not properly polarized, often overlapping each other, and forming disorganized cysts or layers. In addition, distribution of phosphorylated apical myosin II regulatory light chain, which is coupled to activation of actomyosin, is blocked in α-catulin mutants, indicating that α-catulin is required for the apical activation of actomyosin that is necessary for neural tube closure. Adherens junctions are connected to the actomyosin cytoskeleton, and tightly-regulated actomyosin dynamics are critical not only for junction formation, but also for structure and function of adherens junctions during epithelial tissue homeostasis and morphogenesis. Neuroepithelium folding and disorganization phenotype in α-catulin deficient mice could be consistent with the observed changes in P-Mlc2 localization and decreased stability of adherens junctions. Therefore, it would be important in the future to perform laser ablation experiments in order to better understand how α-catulin contributes to the molecular machinery responsible for generating tension at the cell-cell junctions.

Another important aspect of α-catulin deficient phenotype which will require further studies is disorganization of integrin-mediated basement membrane assembly of the α-catulin deficient neuroepithelium, as we visualized by the staining of extracellular matrix components, fibronectin and laminin (Figures 2L,N, arrows in Figures 2L’,N’). Taking into account the complexity of the mouse model, it is possible that the disorganization of basement membrane is the side effect of the lack of cell-cell junction stability or deregulated Rho activity within those cells. Forces generated within cells and tissues are transmitted both through integrin-based adhesions to ECM, and through cadherin-dependent cell–cell adhesions driving tissues morphogenesis. This coordinated regulation of cell–ECM and cell–cell adhesive machinery has emerged as a common theme in a variety of developmental processes; however, its coordination during development is very complex. The ECM ligands can bind to several integrin receptors, and vice versa, generating functional complexity and initiating signaling cascades that are important for cell polarity, regulating cell adhesion and cytoskeletal dynamics (Rozario and DeSimone, 2010; Nikolopoulou et al., 2017). While the importance of cell-cell adhesion has been well characterized in the context of apical constriction, the contribution of cell-ECM adhesion to this process is poorly understood. Recent studies by Fernandes et al., show additional layer of complexity to this process by showing the regulation of integrin expression by signaling pathways. They used the pseudostratified columnar epithelium of the Drosophila eye imaginal disc to explore the connections between upstream signaling pathways, the cytoskeletal response and the contribution of cell-ECM adhesion to the morphogenesis. They showed that Hh and Dpp signaling pathways regulate integrin expression which in turn promote apical constriction by stabilizing microtubules. Thus, integrins genetically linked tissue patterning and morphological change at the cellular level (Fernandes et al., 2014).

Rho GTPases, as crucial factors that regulate actomyosin organization, promote contraction by polymerization of actin via formins or Arp2/3, as well as by promoting myosin light chain (MLC) phosphorylation through activating the myosin light chain kinases MRCK (myotonic dystrophy kinase-related Cdc-42-binding kinase) or Rho kinase (ROCK), which also inhibits myosin phosphatase (MLCP) (Arnold et al., 2017). Rho GTPases cycle between a GTP bound – active form and a GDP bound – inactive form, that is regulated by the presence of activating RhoGEFs (guanine nucleotide exchange factors), and by inactivating RhoGAPs (GTPase activating proteins) (Arnold et al., 2017). Distinct localization of GEFs and GAPs within a cell restricts Rho signaling to a specific region. When misregulated, abnormal junctional F-actin polymerization or myosin II contraction can drive pathological conditions. So it is crucial that this linkage can be dynamically regulated in space and time. Scaffold proteins are a commonly used organization system to bring Rho regulators, GTPases, and effectors together for proper localized Rho GTPase signaling output. We propose that α-catulin acts as a scaffold for proper actin-binding and regulatory protein distribution like active RhoA that plays important functional roles in actin cytoskeleton coordination and cell-cell junctions stabilization. This function of α-catulin, could be mediated through appropriate RhoGEF, as it was shown previously for α-catulin and Lbc-Rho GEF cooperation (Park et al., 2002). An important step to better understand molecular mechanisms of α-catulin function during neurulation will be to characterize tissue specific RhoGEF which would interact with α-catulin.

Using knocked-in beta-galactosidase as a reporter, we showed that expression of α-catulin during early development has a very dynamic and transient pattern in epithelial tissues, which could be correlated with requirement of the temporary presence of this protein, implying that α-catulin could play important roles in mechanotransduction, junctional remodeling, and tissue integrity. Despite a long history of studies of cell-cell junctions, still many unanswered questions remain unanswered, especially in regards to actin’s organization at junctions. It is well established that both Arp2/3 and formins clearly play roles at cell-cell junctions and proper dynamic of elongation and sequestering influence junctions and supports epithelial tension; however, it is not clear how they coordinate to form the junctional actin bundles and generate actin structures which are required during junction remodeling. In the case of NT closure, it will be important to define which formins are involved in this process and if α-catulin, similarly to αE-catenin, could interact directly with formins (Kobielak et al., 2004) to link linear actin nucleation with active Rho function. Recent literature gives such an example, where formin, in this case Fhod3, is critical for proper neurulation by regulating apical constriction (Sulistomo et al., 2019). It is also important to clarify how junctional and medial-apical actomyosin is organized and attached to junctions especially during remodeling and morphogenesis. This could be specific to different tissues, depending upon expression of specific cytoplasmic scaffold proteins that link the transmembrane proteins to actin.

Specific pattern of expression of α-catulin could also be an important piece of information required to build an understanding of the normal and essential functions of that gene and any role it may play in the development or progression of disease. In this case, the expression of α-catulin in the neural crest derived innervation of the intestine supports its possible role in the Hirschsprung disease (HSCR). Hirschsprung disease is a polygenic disorder affecting the enteric nervous system. Loss of innervation in the colon results in defective peristalsis, which causes chronic constipation and abdominal distension. RET is the primary HSCR gene, accounting for 50% of familial cases; however, many modifier loci have been proposed, including two associated regions mapping to chromosome 9q31 which represents a gene-rich location containing four genes, including CTNNAL1 (Tang et al., 2010). In addition, microarray data using mouse knockout of Ret model previously indicated that Ctnnal1 was a gene expressed in the enteric nervous system (Heanue and Pachnis, 2006). Using knocked-in beta-galactosidase as a reporter, we showed strong staining of α-catulin in the peripheral nervous system of the embryos (Supplementary Figures S1B,B’) as well as NB mice (Supplementary Figures S1C,C’,D), including dorsal root ganglia and the enteric nervous system. Our data provide support of the candidacy of Ctnnal1 as an excellent candidate for HSCR modifier.

Developmental biology and cancer biology are the two disciplines that have begun to merge recently, where many cellular activities important for embryogenesis are also key to tumorigenesis (Micalizzi et al., 2010). Cells in order to become more motile disassociate or partially destabilize cadherin/catenin cell-cell contacts through the process called epithelial-mesenchymal transition, either full for single cell migration or partial for collective migration (Thiery, 2002). This event correlates with increased movement and coordination of actomyosin cytoskeleton with cell-cell junctions especially for collective migration. This also requires proper distribution and activation of RhoA. Our study of α-catulin expression during early development showed a very dynamic pattern in epithelial tissues. The expression of α-catulin in adult epithelium is minimal, but is again strongly upregulated and preferentially expressed at the tumor invasion front and in the invasive streams of cells in malignant human head and neck squamous cell carcinomas. Decrease in α-catulin expression in cancer cells results in diminished cell migration and invasion *in vitro* and reduced tumor metastasis *in vivo* in a xenograft mouse model (Cao et al., 2012). This shows how important the role of α-catulin is to both developmental biology and cancer biology. Thus, better understanding of the biologic relevance of α-catulin and its potential downstream signals may shed the light on the coordination of actin-myosin contraction with cell-cell junctions stability that could be common for different biological processes like morphogenetic events, wound healing or cancer progression. This knowledge may lead into better understanding of neural tube related birth defects, and also be valuable in characterization of future therapeutic target for cancer patients.

## Data Availability Statement

The datasets generated for this study are available on request to the corresponding author.

## Ethics Statement

The animal study was reviewed and approved by The University of Southern California Institutional Animal Care and Use Committee.

## Author Contributions

KK contributed to the design of the work, data acquisition, analysis and interpretation, manuscript writing. CC and VY contributed to the data acquisition. MG contributed to the data analysis and interpretation. AK contributed to the design of the work, data acquisition, analysis and interpretation, manuscript writing. All authors approved the submitted version and agreed to be personally accountable for the author’s own contributions and manuscript contents.

## Conflict of Interest

The authors declare that the research was conducted in the absence of any commercial or financial relationships that could be construed as a potential conflict of interest.
