# Supplementary material for: Alpha-Catulin, a New Player in a Rho Dependent Apical Constriction That Contributes to the Mouse Neural Tube Closure
Source: Front Cell Dev Biol. 2020 Mar 17;8:154. doi: 10.3389/fcell.2020.00154 (PMC7089943; doi:10.3389/fcell.2020.00154)
Supplement: Supplementary file 1 [file Data_Sheet_1.PDF]

Supplemental Figure Legends

Figure Sup. 1. Expression pattern of α-catulin at different developmental stages. (A-D) Utilizing the β-galactosidase reporter, we can visualize α-catulin expression pattern at various stages by x-gal staining, co-stained with nuclear fast red for morphology. (A-A'') At E10.5, α-catulin expression is visible in the dermomyotome, around the developing eye, nasal pits, as well as in the developing gut (arrows in A). A' and A'' are cross sections of boxed areas marked in A. (B-B') At 14.5 α-catulin expression is visible in the dorsal root ganglia (DRG) (arrows in B and B'), as well as around the eye, otic vesicle (arrows in B). B' is a cross section of boxed area marked in B. (C – C') In new born (NB) mice α-catulin expression is visible in the DRG (arrows in cross section in upper panel and longitudinal section in lower panel in C). It was additionally confirmed by staining of serial section of catulin lacZ (arrows in upper panel in C') with neuronal marker neurofilament (NF) (arrows in lower panel in C'). (D) Expression of α-catulin is also visible in the innervation of the NB intestine as confirmed by whole mount staining of the intestine with neuronal marker (NF). Magnified area of NB intestine is shown in right panels in D. (E) Confirmation of the correct insertion of the trapped vector in the α-catulin locus by sequencing of cDNA isolated from ES cells before generation of mice. lb-limb bud, nt-neural tube. Asterisks in A' mark DRG. A and B present whole mount stained embryos, whereas D presents whole mount stained intestine.

Figure Sup. 2. (A) Percentage contribution of "good" and "bad" cysts formed by siCTNNAL1 and control (siNeg) MDCK cells in each repetition. Average percentage

contribution from 3 repetitions, SD- standard deviation. (B) Graph showing average percentage contribution of “good” and “bad” cysts formed by siCTNNAL1 and control (siNeg) MDCK cells. $p<0.005$

Figure Sup. 3. (A) Percentage contribution of “good” and “bad” cysts formed by control MDCK cells and α-catulin knock-down (KD1, KD2) MDCK cells in each repetition. SD- standard deviation. (B) Average percentage contribution of “good” and “bad” cysts formed by control MDCK cells and α-catulin knock-down (KD1, KD2) MDCK cells. (C) RT-qPCR analysis of α-catulin level in control MDCK cells and α-catulin knock-down (KD1, KD2) MDCK cells. The results were normalized to GAPDH levels. (D) Graph showing average percentage contribution of “good” and “bad” cysts formed by control MDCK cells and α-catulin knock-down (KD1, KD2) MDCK cells.

Figure Sup. 4. (A) View of cysts appearance formed by control MDCK cells and α-catulin knock-down (KD1, KD2) MDCK cells immunostained with E-cadherin antibody. Arrows indicate “bad” cysts in α-catulin knock-downs. (B) Representative images of cysts formed by control MDCK cells and α-catulin knock-down (KD1, KD2) MDCK cells immunostained with E-cadherin and podocalyxin antibodies, stained with phalloidin and counterstained with DAPI imaged under 63x magnification. Scale bar 10 μm.

Figure Sup. 5. (A) Side view of filter-grown MDCK control cells and α-catulin knock-down (KD1, KD2) MDCK cells immunostained with E-cadherin antibody, stained with phalloidin and counterstained with DAPI. (B) 3D projection of side view of filter-grown MDCK control

cells and α-catulin knock-down (KD1, KD2) MDCK cells immunostained with podocalyxin antibody.

Figure Sup. 6. (A) Schematic representation and WB verification of mouse α-catulin cDNA cloned upstream of GFP into the pEGFPC1 vector. Protein extracts used for WB were isolated from HEK cells (control and transfected with m α-catulinGFP vector). (B) Confocal microscopy analysis under small magnification of filter-grown MDCK cells transfected with α-catulin GFP plasmid and stained with phalloidin. Scale bar 20μm.
