# Supplementary material for: Alpha-Catulin, a New Player in a Rho Dependent Apical Constriction That Contributes to the Mouse Neural Tube Closure
Source: Front Cell Dev Biol. 2020 Mar 17;8:154. doi: 10.3389/fcell.2020.00154 (PMC7089943; doi:10.3389/fcell.2020.00154)
Supplement: Supplementary file 2 [file Data_Sheet_2.PDF]

Supplemental material and methods

Genotyping Primers

Catulin-Ex1-F:	5’ CGGTGCGGGAGCTGTCCACTCC
Catulin-Ex2-R:	5’ CTTGGCTTCGATGCAGGCGAT
BGBR-3:	5’ GACAGTATCGGCCTCAGGAAGATCG

sqRT-PCR Primers

mCatulin-RT-F:	5’ TCGCCTGCATCGAAGCCAAGCAA
mCatulin-RT-R:	5’ ACAGCTGGGATGCCTGAGACATGT
mGAPDH-F:	5’ GCAAAGTGGAGATTGTTGCC
mGAPDH-R:	5’ CCTGCTTCACCACCTTCTTG

Canine specific RT-qPCR primers

canine-catulin Fw4	5’ TGA CTG ACT GCA AAC CAA GTG	product size 246bp
canine-catulin Rev4	5’ GCA ATT CCA GTC TCG TCT GAG	
canine-GAPDH Fw	5’ CAG TTG TGG ATC TGA CCT GC	product size 282bp
canine-GAPDH Rev	5’ CCT TGG AGG CCA TGT AGA CC	

Antibodies for Indirect Immunofluorescence and Immunohistochemistry

**Antibody**	**Dilution**	**Company**
β-Catenin	1:300	Sigma-Aldrich #C2206
ECCD2 (E-cadherin)	1:100	Zymed Laboratories #13-1900
αE-Catenin	1:500	Sigma-Aldrich #C8114
Podocalyxin/gp135	3 μg/ml	DSHB, #3F2/D8 cell line
Fibronectin	1:200	AbCam #ab6328
Keratin 8	1:100	DSHB #TROMA-I
Laminin Ab-1	1:50	Thermo Scientific #RB-082
p-Myosin Light Chain 2 (pMlc2)	1:50	Cell Signaling Technology #3671
Nestin	1:100	DSHB #Rat-401
Neurofilament (NF)	1:300	DSHB #2H3
p75NTR	1:100	Upstate Cell Signaling #07-476
Phalloidin-TRITC	1:200	Fluka #77418
Sox2	1:500	Millipore #AB5603
