# Supplementary figures and images for: Alpha-Catulin, a New Player in a Rho Dependent Apical Constriction That Contributes to the Mouse Neural Tube Closure
Source: Front Cell Dev Biol. 2020 Mar 17;8:154. doi: 10.3389/fcell.2020.00154 (PMC7089943; doi:10.3389/fcell.2020.00154)

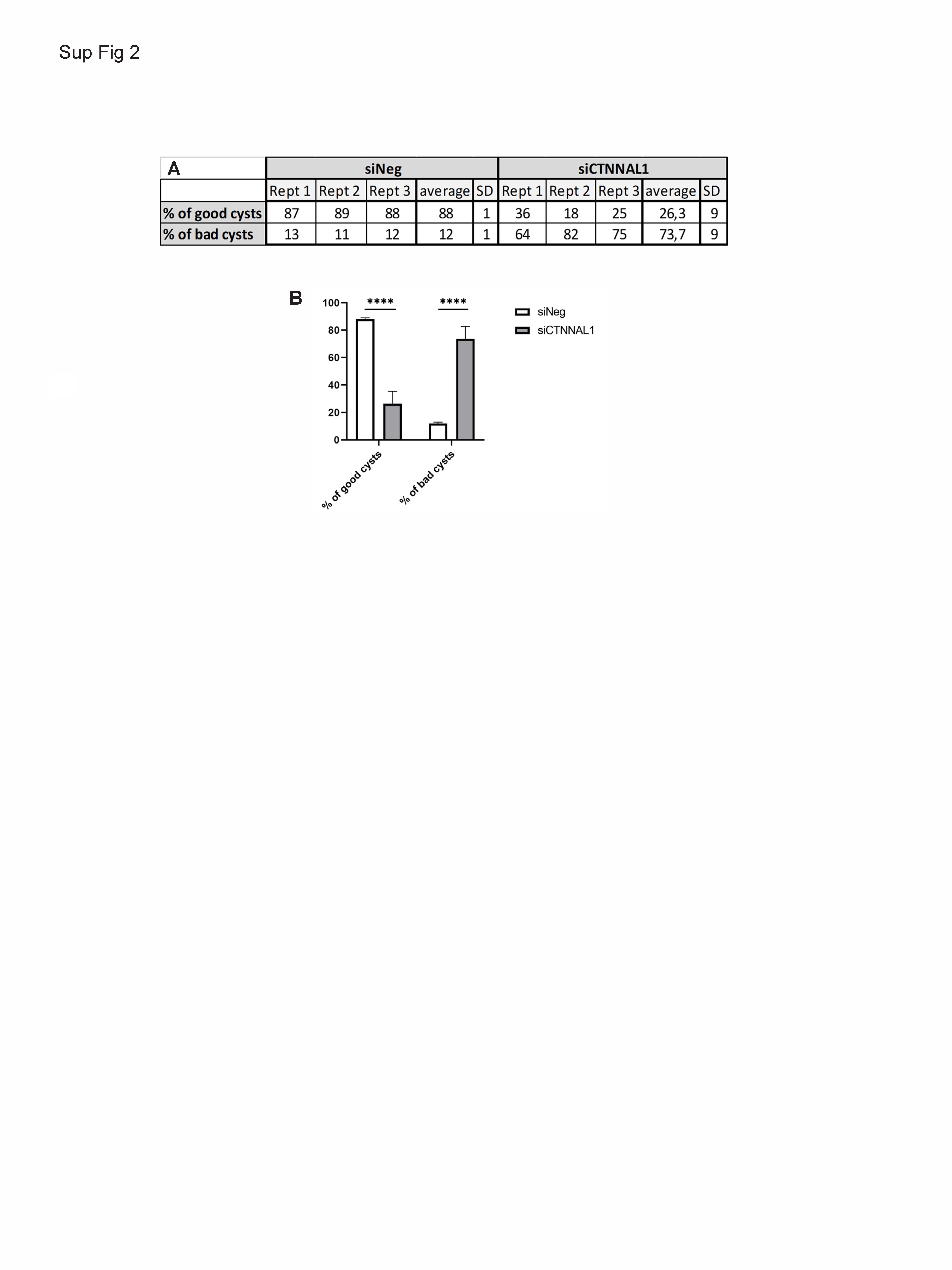

Supplement: Supplementary file 4 [file Image_2.JPEG]

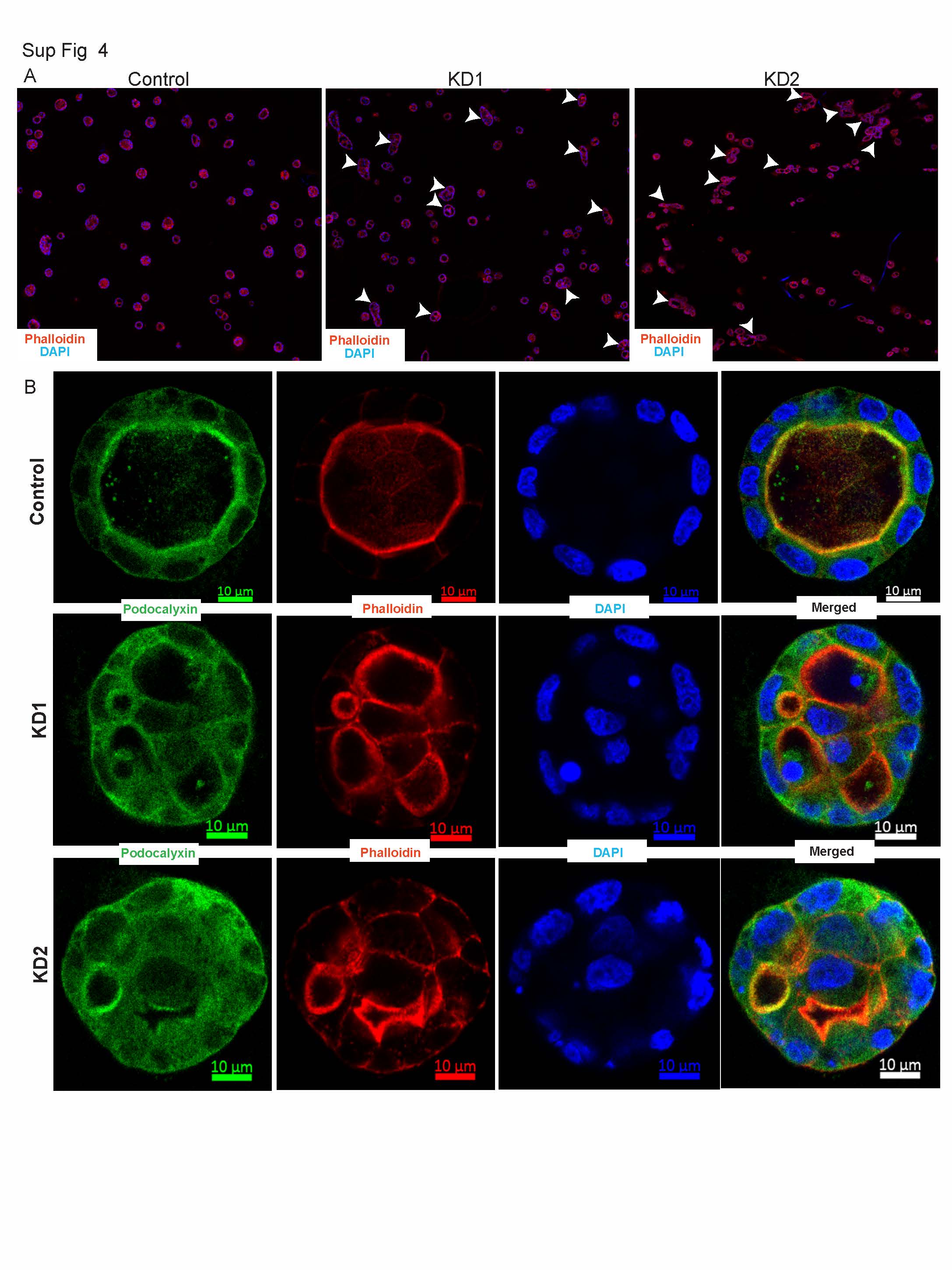

Supplement: Supplementary file 6 [file Image_4.JPEG]

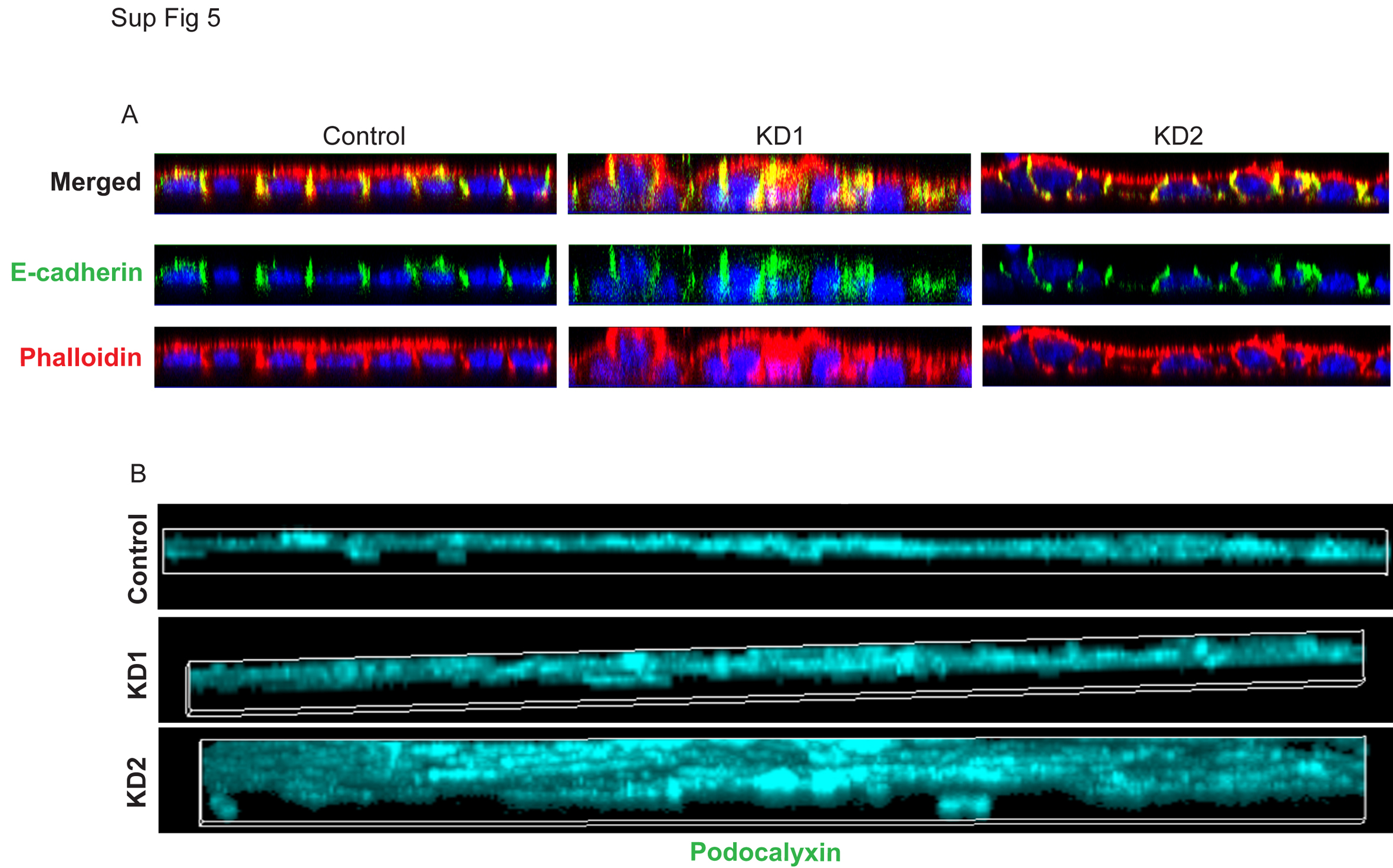

Supplement: Supplementary file 7 [file Image_5.JPEG]

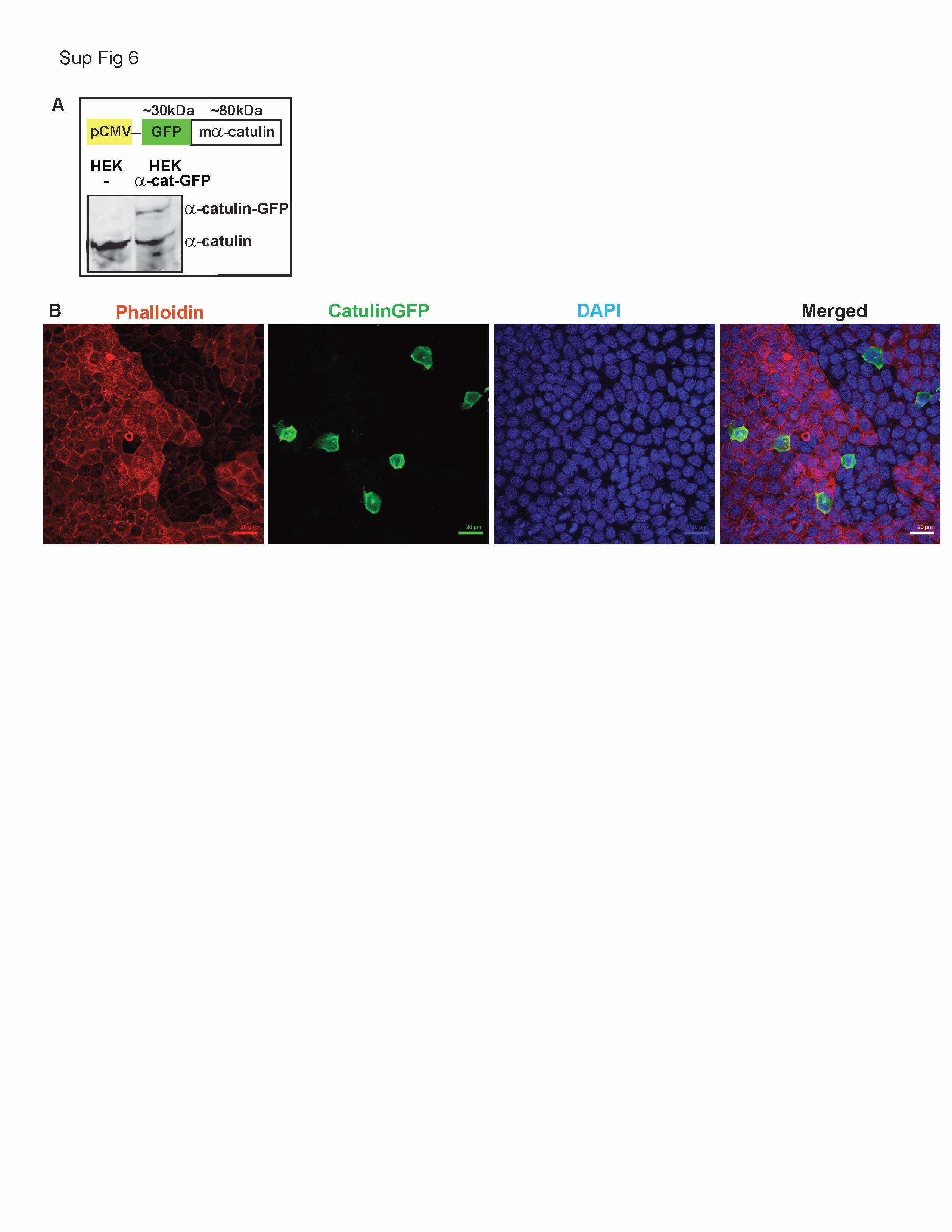

Supplement: Supplementary file 8 [file Image_6.JPEG]
